# Pathways from problems in adolescent family relationships to midlife mental health via early adulthood disadvantages – a 26-year longitudinal study

**DOI:** 10.1371/journal.pone.0178136

**Published:** 2017-05-26

**Authors:** Noora Berg, Olli Kiviruusu, Sakari Karvonen, Ossi Rahkonen, Taina Huurre

**Affiliations:** 1 Department of Public Health Solutions, National Institute for Health and Welfare, Helsinki, Finland; 2 Department of Public Health and Caring Sciences, Uppsala University, Uppsala, Sweden; 3 Department of Health and Social Care Systems, National Institute for Health and Welfare, Helsinki, Finland; 4 Department of Public Health, University of Helsinki, Helsinki, Finland; 5 Department of Health and Social Welfare, City of Vantaa, Vantaa, Finland; Institute of Psychiatry, UNITED KINGDOM

## Abstract

Poor childhood family conditions have a long-term effect on adult mental health, but the mechanisms behind this association are unclear. Our aim was to study the pathways from problematic family relationships in adolescence to midlife psychological distress via disadvantages in early adulthood. Participants of a Finnish cohort study at the age of 16 years old in 1983 were followed up at ages 22, 32 and 42 years old (N = 1334). Problems in family relationships were measured with poor relationship with mother and father, lack of parental support in adolescent’s individuation process and poor home atmosphere, and mental health was assessed using Kessler’s Psychological Distress Scale (K10). We analyzed the indirect effects of adolescent family relations on mental health at age 42 years old via various disadvantages (somatic and psychological symptoms, relationship/marital status, low education/unemployment and heavy drinking) at ages 22 and 32 years old. Problematic adolescent family relationships were associated with midlife psychological distress in women (0.19; 95% CI 0.11, 0.26) and men (0.13; 95% CI 0.04, 0.21). However, after adjustment for adolescent psychological symptoms, the association was only significant for women (0.12; 95% CI 0.04, 0.20). Poor family relationships were associated with various disadvantages in early adulthood. The association from poor family relationships (16 years old) to psychological distress (42 years old) was in part mediated via psychological symptoms in women (0.03; 95% CI 0.01, 0.04) and men (0.02; 95% CI 0.00, 0.04) and in women also via heavy drinking in early adulthood (0.02; 95% CI 0.00, 0.03). Adolescent family relationships have a role in determining adult mental health. Targeted support addressing psychological well-being and hazardous drinking for adolescents with problematic family relationships might prevent disadvantages in early adulthood, and further prevent poor midlife mental health.

## Introduction

Mental health problems account for a major portion of the total burden of disease and are associated with various other disadvantages in life. Several determinants for mental health problems have been identified [1,2], and one of the established associations has been found between social relationships and mental health [3]. Family relationships are usually our primary social relationships in childhood, and the quality of them (including intimacy of relationships, frequency of conflicts and feelings of trust and acceptance [4–6]) form a foundation for a child’s future development.

The results of studies examining the impact of problems in family relationships on adult mental health are not consistent [7]. While some studies have found some features of family relationships to be associated with later poor mental health [8–11], other studies have not found such an association [12], especially after adjusting for gender, age, a person’s current employment, education, income [13] or parental mental health [14]. Longitudinal studies examining this issue are rare and most have followed children or adolescents only to young adulthood at most, and it is still unclear whether the association in question continues to midlife. In one of the rare longitudinal studies with a long follow-up time, Landstedt et al. [15] found an association between poor parental relationships in adolescence and internalizing symptoms at ages 21, 30 and 42 years old, but when the level of internalizing symptoms at age 16 years old was taken into account, the association remained only at age 30 years old. Thus, it might be that previous studies using traditional statistical analyses have not sufficiently taken into account that mental health in adolescence might be part of the pathway to adult mental health. In addition most of the previous studies examining determinants of mental health have studied simple associations between risk factor and later mental health outcome and it is only recently that research on mental health has begun to pay attention to mechanisms and temporal nature of its determinants [16–19]. Risk factors over the life course shape pathways or processes leading to poor mental health. Thus, even if it were not possible to detect a direct association between childhood conditions and midlife wellbeing [20], such associations might transmit indirectly via disadvantages observed at other phases of life [21]. These factors form chains of risks which refer to a sequence of linked exposures where disadvantage in earlier life increases the risk of disadvantage in later phases of life [22], thus linking childhood conditions to adulthood wellbeing. Understanding the underlying mechanisms linking childhood and adulthood wellbeing is important in preventing adverse life careers and poor mental health. Here, we analyze the complex associations between problematic adolescent family relationships and midlife psychological distress.

Parents support their children in various ways, such as by providing physical, human, social and economic capital [23], as well as through normative and behavioral guidance, and social control related to risky behavior, education and social relationships [3]. It might be that the child cannot take advantage of the resources the parents have, if the quality of the family relationships is poor [24]. Poor relationships in the childhood family have been seen as risk factors for the development of poor health [15,25,26], poor social relationships [27–30], low education [31–34], unemployment [35] and risky behavior [36–38], which is most commonly expressed as heavy alcohol use. These factors have been identified as risks for poor mental health [39–44], albeit several other risks have also been identified (pre- and early postnatal environmental adversities, familial psychopathology, stressful life events and severe problems in the family) [1,2,45]. Disadvantage and its accumulation in different dimensions of life might exceed a person’s abilities to cope and adjust to them and can risk mental health later in life [46,47]. Here we, however, focus on these disadvantages that have been found to be associated with both poor family relationships and adulthood mental health problems and the next step would be to examine whether these links shape an indirect pathway from adolescent family relationships all the way to midlife mental health.

The roots of problematic family relationships might be due to adverse life events in the family (e.g. parental divorce, economic adversities), in parental problems (e.g. parental mental health problems) or in factors related to the child (e.g. adjustment problems) [48,49] and these factors can also risk mental wellbeing. In fact severe family adversities such as child abuse and neglect have been seen to represent “the most disturbed end of the spectrum of parent-child relationship quality” [11]. No matter the source, poor relationships can compromise the supportive growth environment of the child. When examining the pathways from childhood or adolescent family to adult mental health, it is important to take widely into account the participant’s living conditions and wellbeing in the beginning of the follow-up in order to elicit the independent effect of family relationships over and above the effects of e.g. child’s mental health status or family’s socioeconomic adversity already in adolescence. It might as well be that poor mental health, risky behaviors or other disadvantages related to the adolescent affect family relationships.

Mixed findings have been found regarding gender differences in the association between problems in family relationships and later mental health, thus further investigation is required. Some studies suggest that girls are more vulnerable to family conflicts than boys [50], while others suggest that boys are more susceptible to family risk factors, at least when mental health is viewed as externalized symptoms, such as conduct problems [49,51], although in some populations no difference has been found [52]. It has also been suggested that the pathways or processes leading to psychological distress are different between genders [46]. Some studies have suggested that women tend to react to stressful life situations more internally, such as with psychological symptoms, while men react more externally, such as with heavy alcohol use [31,53–55], although firm evidence regarding this division remains limited [56]. These inconsistencies have also been explained by suggesting that boys are more vulnerable to family conflicts as younger children, whereas girls are more vulnerable to such conflicts as adolescents [57–59].

**Fig 1:**
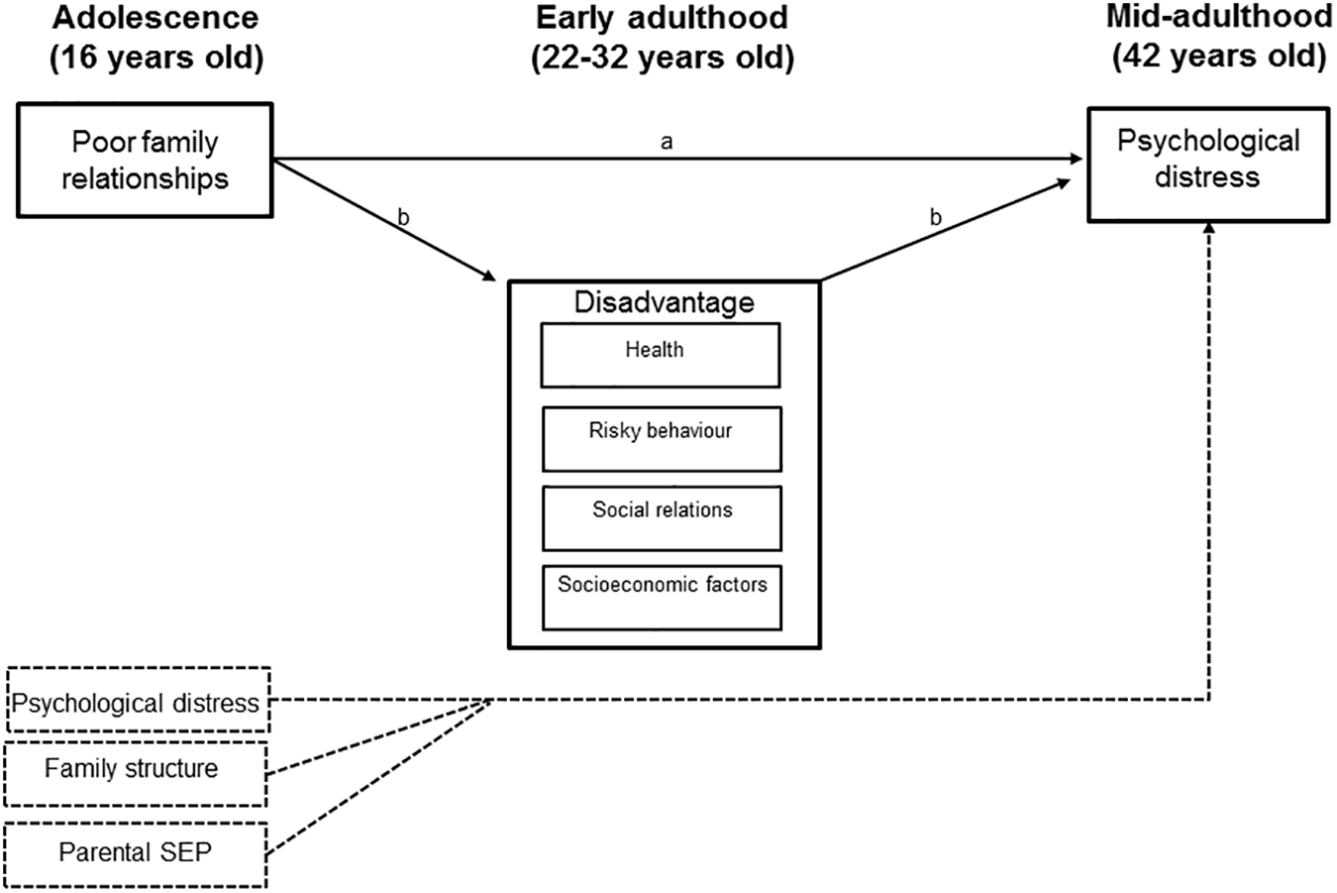
Hypothesized model of the direct (a) and indirect (b) associations between adolescent family relationships and midlife psychological distress, when psychological distress and family structure are taken into account.

Our aims were 1) to study the association between adolescent family relationships and midlife psychological distress and 2) to study the role of various disadvantages in different dimensions of life in early adulthood as mediators of this association. We used a prospective follow-up data spanning 26 years and conducted the study by controlling for adolescent family structure, parental socioeconomic position (SEP), psychological symptoms and other disadvantages. We analyzed disadvantage in several dimensions of life (health, risky behavior, social relations, and socioeconomic factors) simultaneously (Fig 1). We also examined the gender differences in those associations. Previous studies have suggested that boys are more vulnerable to poor family relationships as younger children and girls as adolescents, thus we expected to find stronger associations with midlife psychological distress in women [57].

## Methods

### Data and study population

The original study population included all Finnish-speaking 9^th^ grade pupils attending comprehensive school in Tampere, an industrial and university city of 166 000 inhabitants in southern Finland, in 1983. In the first phase in 1983, 2 194 pupils (96.70% of the target population) with a mean age of 15.92 years (standard deviation (SD) 0.36 years) completed questionnaires during school hours. Follow-ups were conducted using postal questionnaires when the respondents were aged 22 (n = 1656, 75.50%), 32 (n = 1471, 67.00%) and 42 (n = 1334, 60.80%) years old–in 1989, 1999 and 2009, respectively. This cohort had already participated in a brief school study at age 14 years old in 8^th^ grade. Information gathered from this earlier study was available for most (n = 2076, 94.60%) of the participants in this follow-up study. In the present study, only the participants who had participated at age 42 years old were included in the analyses (n = 1334); of these, 86.60% had also participated at age 22 (n = 1155) and 84.20% at age 32 (n = 1123) years old.

In the beginning of 1980s the researches of that time administered the surveys in school classes (ages 14 and 16 years old). They informed the participants of the purposes of the study and that participation was voluntary. Participants indicated their consent by answering the survey questionnaire and in accordance with the prevailing practice consent from the parents was not obtained. The study protocol (including the consent procedure) was approved by the Ethics Committee of Tampere University Hospital. During the follow-ups (ages 22, 32 and 42 years old) information on the study was provided in covering letters and research workers were available to answer any further questions about the study by telephone, email, or mail. The study protocol of the follow-ups was approved by Ethics Committee of Tampere University Hospital at age 22 years old and the Ethics Committee of the National Institute for Health and Welfare (formerly National Public Health Institute) at ages 32 and 42 years old. The participants have had the chance to withdraw from the study at any point. All names and personal identification codes have been removed from the data, thus individual participants cannot be identified by the researchers analyzing the data. However, a small number of authorized personnel have access to the identity data, because identification is necessary for follow-up purposes.

The comparison of participants at age 42 years old follow-up with non-participants showed that non-participants were more likely to be men (60.80% vs. 45.00%, p<0.01), heavy drinkers (25.00% vs. 21.20%, p<0.05) and to have had poorer school grades (mean grade point average 7.34 vs. 7.70, p<0.01) at age 16 years old, although attrition regarding heavy drinking was explained by gender. There were no differences between the participants and the non-participants in problems in family relationships, psychological or somatic symptoms, dating experiences or parental SEP at age 16 years old.

### Measures

#### Problems in family relationships at age 16 years old

For the present study, a comprehensive measure of family relationships was formed, which focused on the perceptions the adolescents had of having problems in warm, caring and supportive interactions with their parents. Problems in family relationships were measured with four indicators measured at age 16 years old: scales of emotional support from the mother and father, a scale of parental support in the adolescent’s individuation process and a single question on the atmosphere at home. The scales of emotional support from the mother and father comprised three statements (e.g. ‘My mother is close to me.’ (reversed)), and the scale of parental support in the adolescent’s individuation process comprised six statements (e.g. ‘Usually my parents trust me.’ (reversed)) [53]. The responses were on a scale from 1 to 5, and the theoretical ranges of the sum scales were 3–15 for the emotional support from the mother/father and 6–30 for parental support in the adolescent’s individuation process, with higher scores indicating problems in support. The scales of emotional support from the mother (Cronbach alpha = 0.71), emotional support from the father (alpha = 0.60) and parental support in the adolescent’s individuation process (alpha = 0.74) showed satisfactory reliabilities. The home atmosphere was reported on a 5-point scale.

#### Psychological distress at age 42 years old

Psychological distress was assessed using the Kessler Psychological Distress Scale (K10) [60]. K10 is a ten-item questionnaire for global measuring of distress based on questions about anxiety and depressive symptoms that a person has experienced in the most recent four-week period. The theoretical range of the scale is 5–50 and Cronbach’s alpha 0.91.

#### Disadvantages at ages 22 and 32 years old

Disadvantages at ages 22 and 32 years old represented four dimensions of life (health, social relations, socioeconomic factors and risky behavior). The somatic and psychological symptoms indicated disadvantages in the health dimension and were measured with a checklist of 17 symptoms [61,62]. The somatic symptoms scale included 12 complaints such as abdominal pain, headache and dizziness. The psychological symptoms scale included five symptoms such as lack of energy or depression and anxiety or nervousness. The respondents reported whether and how often they had had symptoms during the past six months. The social dimension was assessed with being in a relationship (yes/no) (22 years old) and marital status (married or cohabiting vs. other) (32 years old). The socioeconomic dimension was assessed with education (22 years old) and employment status (32 years old). At age 22 years old, the subjects were divided into those who only had a basic education (or at most a short sporadic occupational course) and those who were secondary school graduates or had taken vocational education. At age 32 years old, the subjects were asked about their employment status and were divided into 1) those who were unemployed, temporarily laid off (full-time/part-time), on a disability pension (full-time/part-time) or on an extended sick leave and 2) those who were not included in any of the previous categories. The dimension of risky behavior was measured with heavy drinking. At age 22 years old, frequency of drinking, frequency of being heavily drunk and getting into trouble because of alcohol use during the past 12 months were assessed and compiled as a sum scale of heavy drinking (alpha = 0.61). At age 32 years old, alcohol related problems were assessed with the Alcohol use disorders identification test (AUDIT) [63].

#### Control variables in adolescence

To take into account the baseline levels of psychological distress we used psychological symptoms at age 16 years old as a control variable in the analyses. The psychological symptoms scale at age 16 years old was measured similarly as at ages 22 and 32 years old (see above). Since family relationships might be affected by family structure, we adjusted the models for family structure at age 14 years old with two dichotomous variables indicating reconstituted family (no/yes) and single parenthood (no/yes). For the same reason we adjusted for parental SEP at age 16 years old. It was based on the father’s occupation and if missing, on the mother’s. If neither one’s occupation was available, the parental SEP was based on the parents’ education [31]. Parental SEP was classified into upper non-manual, lower non-manual and manual based on standard classification of occupations [64].

Somatic symptoms at age 16 years old were measured as at ages 22 and 32 years old. School achievement was measured as mean of school marks and classified as good (>7) or poor (≤7) (range 4–10). History of intimate relationships was measured by asking whether the respondent was currently or had ever dated. The measure of heavy drinking was based on frequency of alcohol use and drunkenness at age 16 years old.

### Statistical methods

The analyses were carried out using IBM SPSS Statistics 22 and MPlus 7. For descriptive statistics, means, standard deviations and frequencies of the study variables were reported. First we examined associations between exposure variables (family relationships and disadvantages at ages 22 and 32 years old) and outcome (psychological distress) in univariate and multivariate regression analyses. Then, based on the multivariate model, we selected those variables at ages 22 and 32 years old that were significantly associated (p<0.1) with psychological distress to be used in further analyses.

To analyze the total and indirect effects of problems in family relationships in adolescence on psychological distress in midlife, we used path analyses in the structural equation modelling framework. First, models with only the total effect of problems in family relationships on midlife psychological distress were examined. Then, this model was adjusted for family structure, parental SEP and psychological symptoms in adolescence. In the next step, the disadvantage factors at ages 22 and 32 years old (psychological symptoms, intimate relationship, employment status and heavy drinking) that were associated with outcome were added to the model as intervening variables and indirect effects from the predictor to the outcome via these disadvantages at ages 22 and 32 years old were examined. In additional analyses the indirect effects were controlled for the corresponding variables at age 16 years old (e.g. indirect path via heavy drinking adjusted for heavy drinking at age 16 years old). The Model Indirect option (theta method) in Mplus 7 was used to test the indirect effects, and bootstrapping with 1 000 draws was used to calculate the bias corrected 95% confidence intervals (CI). Because there were endogenous dichotomous variables in the models, we used a weighted least squares means and variance adjusted (WLSMV) estimator in the analyses. We used comparative fit index (CFI), Tucker-Lewis index (TLI) and the root-mean-square error of approximation (RMSEA) to analyze model fit. A multigroup option was used to analyze models in order to obtain separate parameter estimates for women and men, while keeping the measurement model as invariant as possible between genders. Gender differences in the total effects and in the specific indirect paths were tested by constraining the paths to be equal for both genders and by observing significant decrement in model fit as presented by Lau & Cheung [65]. Standardized estimates of the total and indirect effects were reported.

In the models analyzed, family relationships was specified as a latent construct with emotional support from the mother, support from the father, parental support in the adolescent’s individuation process and home atmosphere as its four indicators. The measurement model for family relationships using confirmatory factor analysis showed a good fit to the data after freeing the correlation between the emotional support from the mother and father indicators and allowing separate estimates for women and men for the intercept of the parental support in the adolescent’s individuation process (χ2 = 19.04, df = 7, p < 0.01, CFI = 0.99, TLI = 0.99, RMSEA = 0.05).

## Results

The characteristics of the study subjects are presented in Table 1. In the univariate regression analyses all exposure variables except not being in a relationship at age 22 years old in men were significantly associated with psychological distress at age 42 years old. In multivariate analyses psychological symptoms at ages 22 and 32 years old, not being in a relationship at age 22 years old, unemployment at age 32 years old and heavy drinking at ages 22 and 32 years old were associated with the outcome at least among either gender. Poor family relationships at age 16 years old were no longer associated with age 42 psychological distress when all age 22 and 32 years old variables were analysed in the model simultaneously (Table 2).

**Table 1:** Means and distributions of study variables (N = 1334).

	Women (N = 734)		Men (N = 600)	
	% / mean	(n) / (SD)	% / mean	(n) / (SD)
**Adolescence conditions**				
Poor emotional support from the mother	6.29	(2.70)	6.02	(2.50)
Poor emotional support from the father	6.60	(2.72)	5.89	(2.27)
Poor parental support in the individuation	12.95	(4.75)	13.35	(4.30)
Poor home atmosphere	2.00	(1.10)	1.81	(0.97)
Psychological symptoms 16y	3.46	(2.03)	2.75	(2.03)
Single-parent family 14y	17.50	(124)	13.80	(79)
Reconstituted family 14y	8.80	(62)	8.90	(51)
Parental SEP 16y				
Upper non-manual	17.90	(130)	22.10	(131)
Lower non-manual	32.70	(238)	30.00	(178)
Manual	49.50	(360)	48.80	(285)
**Disadvantages in early adulthood**				
Somatic symptoms 22y	4.84	(3.16)	3.22	(3.08)
Somatic symptoms 32y	5.67	(3.71)	4.52	(3.56)
Psychological symptoms 22y	3.72	(2.22)	2.57	(2.13)
Psychological symptoms 32y	4.22	(2.34)	3.19	(2.21)
Not in a relationship 22y	29.60	(196)	40.40	(199)
Single, divorced, widowed 32y	24.20	(156)	28.10	(133)
Basic education only 22y	13.70	(91)	10.40	(51)
Unemployed^a^ 32y	10.90	(68)	6.80	(31)
Heavy drinking 22y	2.24	(1.65)	3.07	(1.84)
Heavy drinking 32y	4.30	(3.89)	7.67	(5.76)
**Midlife mental health**				
Psychological distress (K10) 42y	14.36	(4.32)	13.61	(4.81)

^a)^ including temporarily laid off (full-time/part-time), disability pensions (full-time/part-time) and extended sick leaves

**Table 2:** Linear univariate and multivariate regression models of the association between poor family relationships, disadvantage at ages 22 and 32 years old and psychological distress at age 42 years old (unstandardized effects).

	Univariate model		Multivariate model	
	Women	Men	Women	Men
	est. (95% CI)	est. (95% CI)	est. (95% CI)	est. (95% CI)
Poor family relationships age 16	0.36 (0.21, 0.52)	0.31 (0.09, 0.53)	0.04 (-0.11, 0.18)	0.04 (-0.20, 0.28)
Somatic symptoms age 22	0.35 (0.25, 0.45)	0.37 (0.25, 0.48)	0.08 (-0.04, 0.20)	0.02 (-0.15, 0.20)
Somatic symptoms age 32	0.36 (0.28, 0.44)	0.47 (0.36, 0.58)	-0.05 (-0.16, 0.06)	0.07 (-0.09, 0.22)
Psychological symptoms age 22	0.73 (0.59, 0.86)	0.70 (0.54, 0.87)	0.21 (0.04, 0.38)	0.25 (0.00, 0.50)^a^
Psychological symptoms age 32	0.84 (0.72, 0.96)	0.93 (0.77, 1.10)	0.65 (0.48, 0.82)	0.57 (0.32, 0.82)
Not in a relationship age 22	1.23 (0.52, 1.94)	0.40 (-0.37, 1.17)	0.60 (-0.05, 1.25)^a^	0.06 (-0.78, 0.90)
Single/divorced/widowed age 32	0.91 (0.17, 1.65)	1.97 (1.07, 2.88)	0.22 (-0.48, 0.91)	0.80 (-0.16, 1.76)
Low education age 22	1.60 (0.66, 5.54)	1.51 (0.28, 2.74)	0.20 (-0.69, 1.08)	-0.05 (-1.42, 1.33)
Unemployment age 32	2.86 (1.84, 3.87)	3.21 (1.55, 4.86)	1.58 (0.65, 2.51)	1.74 (0.04, 3.45)
Heavy drinking age 22	0.24 (0.04, 0.44)	0.31 (0.11, 0.51)	-0.21 (-0.42, 0.00)^a^	-0.01 (-0.26, 0.24)
Heavy drinking age 32	0.28 (0.20, 0.36)	0.20 (0.13, 0.27)	0.15 (0.06, 0.24)	0.05 (-0.04, 0.13)

^a^ Significant at 90% CI

Next, we examined the total effects of family relationships on the midlife psychological distress in more detail. The fit indices of the unadjusted model were χ2 = 29.01, df = 13, p < 0.01, RMSEA = 0.04, CFI = 0.99, TLI = 0.98, indicating a good fit to the data. Problems in family relationships in adolescence were associated with psychological distress in midlife for women and men (path coefficients 0.19, 95% CI (0.11, 0.26) and 0.13, 95% CI (0.04, 0.21) respectively), but after adjusting for psychological symptoms in adolescence (0.12, 95% CI (0.04, 0.20), 0.05, 95% CI (-0.05, 0.15)), this was no longer the case for men. Adjusting also for family structure and parental SEP in adolescence did not change the effects significantly (0.11, 95% CI (0.03, 0.19), 0.05, 95% CI (-0.05, 0.15)). This gender difference was not statistically significant.

**Fig 2:**
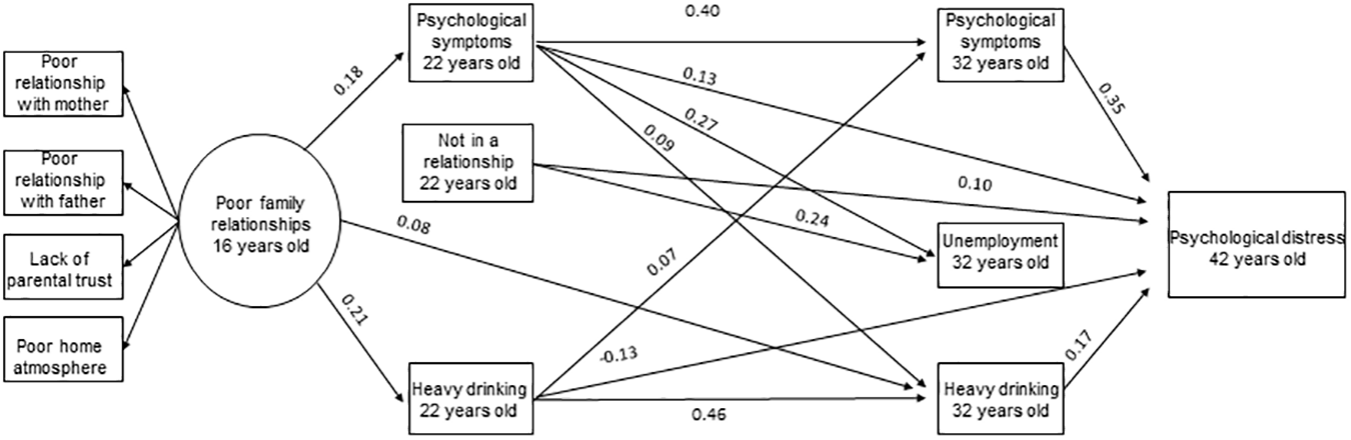
Significant (95% CI) associations between family relationships in adolescence, disadvantage factors at ages 22 and 32 years old and mental health in midlife in women. **The model is adjusted for family structure (14 years old), parental SEP (16 years old) and psychological symptoms (16 years old).** Note: Significant correlations between factors at age 22 years old and between factors at age 32 years old are not shown in order for the figure to remain clear.

**Fig 3:**
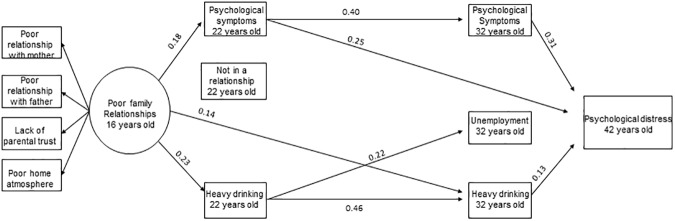
Significant (95% CI) associations between family relationships in adolescence, disadvantage factors at ages 22 and 32 years old and mental health in midlife in men. **The model is adjusted for family structure (14 years old), parental SEP (16 years old) and psychological symptoms (16 years old).** Note: Significant correlations between factors at age 22 years old and between factors age 32 years old are not shown in order for the figure to remain clear.

Then, all the disadvantage variables at ages 22 and 32 years old that were in previous analyses found to be significantly associated with the outcome in either men or women were included in the model simultaneously. The model showed a good fit (χ2 = 116.55, df = 73, p<0.01, RMSEA = 0.03, CFI = 0.98, TLI = 0.96). Significant path coefficients (95% CI) between the variables are illustrated in Fig 2 for women and in Fig 3 for men. In women, poor adolescent family relationships were associated with more psychological symptoms and heavy drinking at age 22 years old. In addition to psychological symptoms at ages 22 and 32 years old, heavy drinking at ages 22 and 32 years old and not being in a relationship at age 22 years old were associated with psychological distress at age 42 years old in women. In men, poor family relationships were associated with psychological symptoms and heavy drinking at age 22 years old. Psychological symptom variables (22 and 32 years old) and heavy drinking at age 32 years old were associated with age 42 years old psychological distress in men. Psychological symptoms and heavy drinking at age 22 years old were associated with the corresponding disadvantage at age 32 years old, but also some associations across different disadvantages were found, especially in women.

Finally the indirect effects were examined and Table 3 summarizes the indirect effects when all these disadvantage factors were analyzed simultaneously in one model. The sum of indirect effects (i.e. all specific indirect effects combined) was significant, indicating that the intermediate factors (psychological symptoms 22 and 32y, not being in a relationship 22y, heavy drinking 22 and 32y and unemployment 32y) had a significant role in transmitting the association between problems in family relationships and psychological distress. Inspection of the specific paths revealed that problems in family relationships in adolescence and midlife psychological distress were associated through psychological symptoms (22y and 22-32y) for both genders. For women the association went also via heavy drinking (22y and 22-32y). Gender differences in these indirect effects were not statistically significant. We conducted an additional analysis (Table 3, Model 2) to examine more precisely this indirect effect when heavy drinking at age 16 years old was taken into account. In this analysis, the indirect pathways remained, although via heavy drinking at age 22 years old in women attenuated to marginally significant (90% CI).

**Table 3:** Indirect effects of problems in family relationships (16y) on psychological distress (42y) through disadvantages (22, 32y).

Problems in family relationships	Model 1^a^		Model 2^b^	
	est.	(CI 95%)	est.	(CI 95%)
**Women**				
Sum of indirect effect	**0.09**	**(0.03,0.15)**	**0.08**	**(0.02,0.14)**
**Paths via**				
Psychological symptoms 22y	**0.02**	(0.00,0.05)*	**0.02**	(0.00,0.04)*
Psychological symptoms 32y	0.00	(-0.03, 0.03)	-0.01	(-0.04, 0.03)
Not in a relationship 22y	0.01	(-0.01, 0.03)	0.01	(-0.01, 0.03)
Unemployment 32y	0.01	(-0.02, 0.03)	0.01	(-0.02, 0.03)
Heavy drinking 22y	**-0.03**	(-0.05,-0.00)*	-0.02	(-0.04,0.00)
Heavy drinking 32y	0.02	(0.00, 0.03)	0.01	(-0.01, 0.03)
Psychological symptoms 22y and 32y	**0.03**	**(0.01,0.04)**	**0.03**	**(0.01,0.04)**
Not in a relationship 22y and psychological symptoms 32y	0.00	(0.00, 0.01)	0.00	(0.00, 0.01)
Heavy drinking 22y and psychological symptoms 32y	0.01	(0.00,0.01)	0.00	(0.00,0.01)
Psychological symptoms 22y and unemployment 32y	0.01	(0.00,0.01	0.01	(0.00,0.01)
Not in a relationship 22y and unemployment 32y	0.00	(0.00, 0.01)	0.00	(0.00, 0.01)
Heavy drinking 22y and unemployment 32y	0.00	(-0.01, 0.00)	0.00	(0.00, 0.00)
Psychological symptoms 22y and heavy drinking 32y	0.00	(0.00, 0.01)	0.00	(0.00, 0.01)
Not in a relationship 22y and heavy drinking 32y	0.00	(0.00,0.00)	0.00	(0.00,0.00)
Heavy drinking 22y and 32y	**0.02**	(0.00,0.03)*	**0.01**	(0.00,0.03)*
**Men**				
Sum of indirect effect	**0.09**	(0.00,0.18)*	**0.10**	**(0.01,0.19)**
**Paths via**				
Psychological symptoms 22y	**0.05**	**(0.01,0.08)**	**0.05**	**(0.01,0.10)**
Psychological symptoms 32y	-0.01	(-0.05, 0.03)	-0.01	(-0.06, 0.04)
Not in a relationship 22y	0.00	(-0.01, 0.01)	0.00	(-0.01, 0.02)
Unemployment 32y	0.01	(-0.03, 0.04)	0.01	(-0.03, 0.04)
Heavy drinking 22y	-0.01	(-0.04,0.02)	-0.01	(-0.03,0.02)
Heavy drinking 32y	0.02	(-0.01, 0.05)	0.02	(-0.01, 0.05)
Psychological symptoms 22y and 32y	**0.02**	(0.00,0.04)*	**0.03**	**(0.01,0.05)**
Not in a relationship 22y and psychological symptoms 32y	0.00	(-0.01, 0.01)	0.00	(-0.01, 0.01)
Heavy drinking 22y psychological symptoms 32y	0.00	(-0.01,0.01)	0.00	(-0.01,0.01)
Psychological symptoms (22y) and unemployment (32y)	0.00	(-0.00,0.01)	0.00	(-0.00,0.01)
Not in a relationship 22y and unemployment 32y	0.00	(-0.00, 0.00)	0.00	(-0.00, 0.00)
Heavy drinking 22y and unemployment 32y	0.00	(-0.01, 0.01)	0.00	(-0.01, 0.01)
Psychological symptoms 22y and heavy drinking 32y	0.00	(-0.00, 0.00)	0.00	(-0.00, 0.00)
Not in a relationship 22y and heavy drinking 32y	0.00	(-0.00,0.00)	0.00	(-0.00,0.00)
Heavy drinking 22y and 32y	0.01	(-0.01,0.03)	0.01	(-0.01,0.02)

^a^ Model 1 Adjusted for adolescent family structure, parental SEP and psychological symptoms

^b^ Model 2 Model 1 + adjusted for heavy drinking

* Figures have been rounded and do not include zero

## Discussion

The aim of this study was to examine contributions of several early adult disadvantages in shaping the association between problems in family relationships in adolescence and psychological distress in midlife. We found indirect effects via early adult poor mental health in both genders and via heavy drinking in women, although gender differences were not statistically significant. Few studies have examined the indirect effects of problems in adolescent family relationships on midlife mental health via early adult disadvantages.

In previous studies, two issues have been raised: whether there is an association between childhood family conflicts (milder than severe adversities) and adult mental health [7], and whether there are gender-specific differences in this association [66]. We found an association between poor adolescent family relationships and midlife psychological distress in both genders, but when psychological symptoms in adolescence were taken into account, this association remained only for women. This is in line with the perception that problems in family relationships in adolescence are more strongly associated with later mental health problems for girls than for boys [57]. This has been explained by evolving gender roles [9,57]. It has been suggested that because girls are socialized into valuing interpersonal relationships more than boys, they are more preoccupied with maintaining good relationships and may thus be more adversely affected by family conflicts [66,67]. It might be that in men, mental health is more strongly determined by other factors.

It is difficult to compare our results with those of previous studies because of diversity in methodology, especially in defining exposures and outcomes. Mixed findings related to the existence of an association between family relationships and mental health and gender differences in this association have been found in previous studies. These differences in findings might be due to the timing of the measurement of family relationships as Davies & Windle [57] suggest, i.e. that the effect is more pronounced in adolescence for girls, but for boys more likely to be observed earlier in childhood. Mixed findings may also be due to variations in adjustments as well as a lack of thorough analyses of gender differences (not just adjusting for gender).

Previous studies have shown that problems in family relationships are associated with disadvantages in many dimensions of life, but often these associations have been examined separately. As Dannefer et al. [68] point out, the life course pathways actually form a complex network. Thus, it is only by analyzing the pathways simultaneously that we can investigate the multidimensional nature of this phenomenon and how the pathways relate to each other. In line with the chain of risk model [22], we hypothesized that poor family relationships would act as a risk factor for many subsequent disadvantages in early adulthood that would further act as risk factors for midlife psychological distress. We found indirect effects via psychological symptoms and heavy drinking. The association from poor family relationships to psychological distress was transmitted via psychological symptoms in early adulthood in both genders. This result contributes to explaining our findings about gender difference in the total effect described previously. Family relationships were associated with midlife mental health also in men, but this path to could only be detected when the indirect effect via mental health in early adulthood was examined. In fact, this age 16 years old adjusted insignificant total effect could suggest that in men the role of psychological symptoms at age 16 years old is more likely to be a part of the mediation path from family relationships to midlife distress rather than just a control variable. In men, the effect is fully mediated, while in women this similar mediation pathway explains the total effect only partially. Poor family relationships might directly increase psychological distress in adolescence which further continues to midlife. Moreover, poor family relationships could also compromise the development of the social competencies and social skills of the adolescent, which further increases the risk of psychological problems [69].

The association between poor family relationships and midlife distress also passed through heavy drinking in women. This path through heavy drinking was not found in men, although the gender difference was not statistically significant. The path in women remained after adjusting for heavy drinking at age 16 years old. Adolescence is often the time of first experiences with alcohol, and other substances, and this can be a common subject of conflicts in the family. If parents can deal with these conflicts with the adolescent constructively and, despite these conflicts, continue to have open and supportive relationships with the adolescent, it might be that this problematic heavy drinking does not extend to adulthood. However, heavy drinking might also be a way to self-medicate oneself to ease the burden of troubled family relationships [70]. It has been suggested that low parental support could be associated with adolescent alcohol use via emotional and physiological arousal in response to family interactions [71]. Whatever the exact chain of effects, our results suggest that heavy alcohol use is one mechanism through which poor family relationships exert their influence on adulthood mental well-being, and as such possibly provide a focal point for intervention efforts.

In the analyses of attrition, the non-participants were more likely to be men and heavy drinkers, which may have weakened the association via heavy drinking for men. In addition, the total effect of family relationships on midlife distress was not as strong for men as it was for women, which might have weakened the indirect effects as well. Thus, strong conclusions about gender differences cannot be made. Further, these results do not support the idea of women reacting to poor family relationships internally with psychological symptoms or men externally with heavy drinking, as both of these disadvantages were associated with poor adolescent family relationships in both genders.

We examined several possible indirect pathways, but only few were found. Considering the long follow-up time and that support from friends and intimate partners usually replaces or at least supplements the parental support when adolescents become adults, it is interesting to find that adolescent family relationships have such longstanding effects as far as to midlife. Although, mainly the associations found were between the corresponding age 22 and 32 disadvantages, some associations across different disadvantages were found especially in women. These associations between different disadvantages suggest that the pathways from poor family relationships form a complex network of disadvantages through the life course. For example, psychological symptoms at age 22 years old were associated with several disadvantages at age 32 years old in women (Fig 2). Also the statistically significant sum of indirect effects supports this idea of a complex web of associations and suggests that these disadvantages together mediate the association with psychological distress. Thus, the indirect effect of family relationships on midlife mental health is scattered via so many different disadvantages that they are hard to detect independently. Our results suggest that the problems in family interactions they face as adolescents can lead to problems in several dimensions of life. Furthermore, these problems or disadvantages can cause a psychological burden. Rather than emphasizing certain specific indirect effects studied in isolation, our results suggest a more multidimensional approach, in which all these factors are associated simultaneously as a network.

It is not possible to make firm conclusions about the direction of the associations. Poor family relationships at age 16 years old might reflect problems in mental health and risky behavior already at that age phase. Therefore, it is not certain that problems in family relationships preceded problems in different dimensions of life. We took into account family structure and resources (SEP), mental health and heavy drinking in adolescence in the analyses and although the total effect attenuated for men, the indirect paths via mental health and heavy drinking (for women) in early adulthood remained relevant. Thus, while the disadvantage already at age 16 years old impacts the indirect pathways from problems in family relationships to psychological distress in midlife, the family relationships continue to have a unique role.

### Methodological considerations

The main strengths of the present study are a long prospective follow-up time and a multidimensional approach to disadvantage. However, when interpreting the results, a few methodological issues should be remembered. There was attrition regarding male gender and poor school achievement. Therefore it is possible that pathways in men and regarding education/unemployment could differ from those found in this study. Eerola et. al [72] have concluded that attrition did not seriously bias the estimation of depression prevalence at age 32 years old in this dataset. Thus, this is likely the case also regarding other mental health measures (K10 in the present study). All the measures were self-reports and thus are prone to the general problems of self-reporting. Especially, regarding family relationships it is a limitation that we only had information from the participants and not from their parents or other family members, although it has been suggested that there is only moderate variation in the parent’s and adolescent’s evaluation of the interactions [73]. Although we were able to use a comprehensive measure of family relationships—which is an advantage especially when measuring adolescent relationships–it was not a standardized measure. The psychological distress measure (K10) has been shown to screen for serious mental disorders effectively [60]. Furthermore, we were not able to measure the duration of problems in family relationships or of other problems. Previous studies have shown that the longer a stressful situation lasts, the stronger its impact will be later in life [74]. Also the time between the follow-ups was rather long (6–10 years), which might have attenuated the associations and in part explain why the sizes of indirect effects were rather modest.

The aim of this study was to examine pathways from adolescent family relationships to midlife psychological distress using a multidimensional perspective; however, there are other relevant factors that probably affect these pathways that were not taken into account, such as genetic and personal characteristics, pre- and postnatal environmental adversities and life events. In addition, social structures, psychosocial resources and human agency all have a role in shaping the pathways of disadvantage and wellbeing [75].

## Conclusions

Family relationships in adolescence are associated with midlife mental health. Mental health in early adulthood steers this association. Also heavy drinking plays a role in it. It is important to support families with problematic relationships, because a lack of warm and supporting family relationships in adolescence could compromise successful pathways to adult wellbeing. Especially interventions developed for improving interaction with children and parents could also prevent mental health problems in early adulthood and adulthood. In addition to psychological distress, our results highlight alcohol use as a probable effective target for interventions.
